# Supplementary material for: Venomix: a simple bioinformatic pipeline for identifying and characterizing toxin gene candidates from transcriptomic data
Source: PeerJ. 2018 Jul 31;6:e5361. doi: 10.7717/peerj.5361 (PMC6074769; doi:10.7717/peerj.5361)
Supplement: Supplemental Information 6 [file peerj-06-5361-s006.gz › FinalOutput_1E-6/Zinc_metalloproteinase@disintegrin_107/finaltree.pdf]

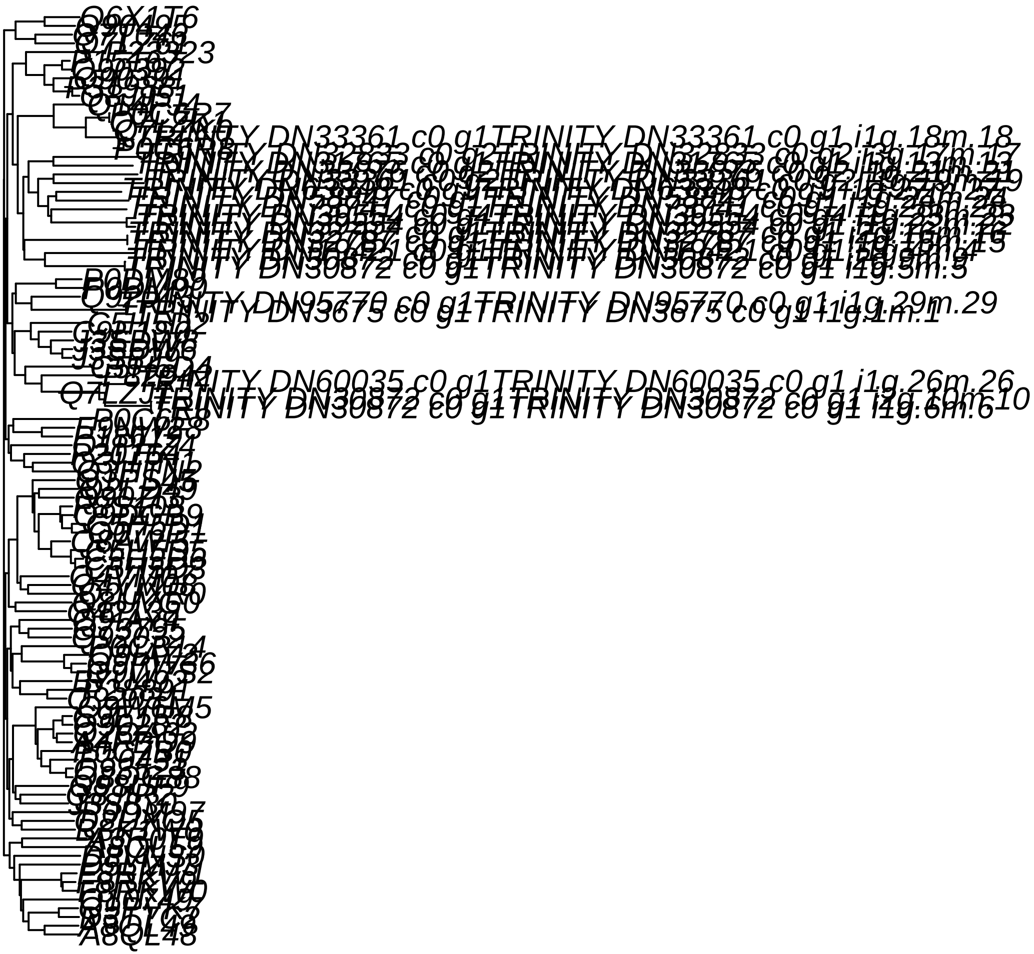
Q6X1T6
TRINITY DN33361 c0 g1TRINITY DN33361 c0 g1 i1g.18m.18
TRINITY DN30872 c0 g1TRINITY DN30872 c0 g1 i1g.5m.5
TRINITY DN95770 c0 g1TRINITY DN95770 c0 g1 i1g.29m.29
TRINITY DN3675 c0 g1TRINITY DN3675 c0 g1 i1g.1m.1
TRINITY DN60035 c0 g1TRINITY DN60035 c0 g1 i1g.26m.26
TRINITY DN30872 c0 g1TRINITY DN30872 c0 g1 i1g.6m.6
A8QL48
